# Biomarkers and Bacterial Pneumonia Risk in Patients with Treated HIV Infection: A Case-Control Study

**DOI:** 10.1371/journal.pone.0056249

**Published:** 2013-02-15

**Authors:** Sonja M. Bjerk, Jason V. Baker, Sean Emery, Jacqueline Neuhaus, Brian Angus, Fred M. Gordin, Sarah L. Pett, Christoph Stephan, Ken M. Kunisaki, for the INSIGHT SMART Study Group

**Affiliations:** 1 Division of Pulmonary, Allergy, Critical Care and Sleep Medicine, University of Minnesota, Minneapolis, Minnesota, United States of America; 2 Division of Infectious Diseases, Hennepin County Medical Center, Minneapolis, Minnesota, United States of America; 3 The Kirby Institute, University of New South Wales, Sydney, New South Wales, Australia; 4 Division of Biostatistics, University of Minnesota, Minneapolis, Minnesota, United States of America; 5 Nuffield Department of Medicine, University of Oxford, Oxford, United Kingdom; 6 Division of Infectious Diseases, Washington D.C. Veterans Affairs Medical Center, Washington, District of Columbia, United States of America; 7 Division of Infectious Diseases, George Washington University, Washington, District of Columbia, United States of America; 8 HIV/Immunology and Infectious Diseases Clinical Services Unit, St. Vincent’s Hospital, Sydney, New South Wales, Australia; 9 Division of Infectious Diseases, Johann Wolfgang Goethe-University Hospital, Frankfurt, Germany; 10 Minneapolis Veterans Affairs Health Care System, Minneapolis, Minnesota, United States of America; National Institute of Infectious Diseases, Japan

## Abstract

**Background:**

Despite advances in HIV treatment, bacterial pneumonia continues to cause considerable morbidity and mortality in patients with HIV infection. Studies of biomarker associations with bacterial pneumonia risk in treated HIV-infected patients do not currently exist.

**Methods:**

We performed a nested, matched, case-control study among participants randomized to continuous combination antiretroviral therapy (cART) in the Strategies for Management of Antiretroviral Therapy trial. Patients who developed bacterial pneumonia (cases) and patients without bacterial pneumonia (controls) were matched 1∶1 on clinical center, smoking status, age, and baseline cART use. Baseline levels of Club Cell Secretory Protein 16 (CC16), Surfactant Protein D (SP-D), C-reactive protein (hsCRP), interleukin-6 (IL-6), and d-dimer were compared between cases and controls.

**Results:**

Cases (n = 72) and controls (n = 72) were 25.7% female, 51.4% black, 65.3% current smokers, 9.7% diabetic, 36.1% co-infected with Hepatitis B/C, and 75.0% were on cART at baseline. Median (IQR) age was 45 (41, 51) years with CD4+ count of 553 (436, 690) cells/mm^3^. Baseline CC16 and SP-D were similar between cases and controls, but hsCRP was significantly higher in cases than controls (2.94 µg/mL in cases vs. 1.93 µg/mL in controls; p = 0.02). IL-6 and d-dimer levels were also higher in cases compared to controls, though differences were not statistically significant (p-value 0.06 and 0.10, respectively).

**Conclusions:**

In patients with cART-treated HIV infection, higher levels of systemic inflammatory markers were associated with increased bacterial pneumonia risk, while two pulmonary-specific inflammatory biomarkers, CC16 and SP-D, were not associated with bacterial pneumonia risk.

## Introduction

Globally in 2010, approximately 34 million persons were living with human immunodeficiency virus (HIV) infection and an estimated 1.8 million people died of acquired immunodeficiency syndrome (AIDS) [1]. Effective combination antiretroviral therapy (cART) has greatly improved life expectancy and decreased the incidence of Pneumocystis pneumonia [2] and bacterial pneumonia [3]. However, community-acquired bacterial pneumonia continues to present as a major clinical problem.

Highlighting the scale of problem is the observation made in the Strategies for Management of Antiretroviral Therapy (SMART) trial [4], where 65% of the adverse clinical events were non-AIDS related and single episodes of bacterial pneumonia were the most frequent clinical event at 2.1% (during a mean follow-up time of 16 months) [5]. A cohort study of US women reported the rate of bacterial pneumonia at 8.5 per 100 person-years in HIV-infected patients, a 12-fold increase compared to a rate of 0.7 per 100 person-years in HIV-uninfected patients [6]. Importantly, bacterial pneumonia in that study was also associated with faster time to death, even after adjustment for CD4+ count and cART use. A Danish cohort study also reported that hospitalization for bacterial pneumonia was associated with an increased risk of death, even more than one year after the hospitalization [3]. Bacterial pneumonia in HIV-infected patients is also associated with permanent declines in lung function [7], more airflow obstruction [8], and a higher risk of lung cancer [9].

Major risk factors for bacterial pneumonia in patients with HIV infection include lower CD4+ count, higher viral load, older age, cigarette smoking, lack of cART, interruption of cART, alcohol use, injection drug use, and lack of pneumococcal vaccination [5], [6], [10]–[14]. Despite the significant impact of bacterial pneumonia in HIV-infected patients, there are currently no additional tools to predict bacterial pneumonia risk in HIV infection. In addition, the mechanisms by which HIV increases bacterial pneumonia risk, once effective treatment with cART is started, are unknown.

Patients with HIV infection demonstrate abnormal chronic pulmonary inflammatory responses such as a CD8+ T-cell alveolitis in bronchoalveolar lavage fluid [15]. We hypothesized that this abnormal pulmonary inflammation may play a role in the higher bacterial pneumonia risk observed in patients with HIV infection. We further hypothesized that such relationships would be independent of the systemic inflammation often observed in patients with HIV infection [16]. We therefore examined relationships between biomarkers, both lung-specific and non-organ-specific, and bacterial pneumonia events among a cohort of patients with treated HIV infection.

## Methods

### Study Population

We performed a nested, matched case-control study, using previously collected data and stored blood samples from the SMART trial (ClinicalTrials.gov identifier NCT00027352) [4]. The SMART trial established the superiority of a strategy of continuous cART compared to intermittent cART.

Eligibility criteria for the SMART trial included a CD4+ count greater than 350 CD4+ cells/mm^3^, and an age greater than 13 years. Within the SMART cohort, 204 participants experienced a bacterial pneumonia event–82 in the continuous cART arm and 122 in the intermittent cART arm. We examined the current clinically relevant question of whether biomarkers could help determine bacterial pneumonia risk in the continuous cART arm, which represents the current standard of providing continuous cART once cART is started. Therefore, cases were defined as patients who developed bacterial pneumonia in the continuous cART arm of SMART. Controls were selected among SMART participants in the continuous cART arm who did not develop bacterial pneumonia (case:control ratio of 1∶1) matched on clinical center, smoking status, age ±10 years, and baseline cART use.

The SMART trial was approved by each study center’s local institutional review board and/or ethics committee, all patients provided written informed consent using procedures approved by local institutional review boards and/or ethics committees, and documentation of informed consent was confirmed by the central statistical and data management center prior to study enrollment. Participants in this secondary analysis also provided written informed consent for storage and future analysis of their biologic specimens for analyses such as this current study (see acknowledgement section for a complete list of study centers from which participants in this secondary analysis were selected). Written informed consent was obtained from next of kin, caretakers, or legal guardians on the behalf of the minors and children in the trial.

### Data Collection and Follow-up

Before randomization, participants received a targeted physical examination and had their medical history taken. Baseline data included history of smoking (current, past, or never) and injection-drug use. Participants were asked at baseline about their history of prior AIDS-defining events, including history of prior/recurrent bacterial pneumonia. No information was collected about immunizations or about the routine use of antibiotics, including co-trimoxazole as opportunistic infection prophylaxis. Follow-up study visits were scheduled at 1 and 2 months after randomization, then every 2 months for the first year, and then every 4 months for the remainder of the study.

During the study, a standardized case report form was used to collect information on patients with suspected bacterial pneumonia. Patients were evaluated for pneumonia using local clinical standards and guidelines. A central clinical events committee, blinded to study arm, evaluated each report and classified bacterial pneumonia events into one of three categories: (1) “confirmed” (compatible clinical and radiographic evidence with histologic or microbiologic support); (2) “probable” (signs and symptoms of pneumonia with compatible radiographic abnormalities); or (3) “suspected” (signs or symptoms of pneumonia with no supporting radiographic evidence). In this report, we include data on patients with bacterial pneumonia that was classified by the clinical endpoint committee as “confirmed” or “probable.”

### Biomarker Measurements

In order to test our hypothesis regarding the relationship of baseline blood biomarkers of inflammation and subsequent bacterial pneumonia events, we chose to evaluate five biomarkers, two of which are felt to be specific for pulmonary inflammation–Club (Clara) cell protein 16 (CC16) and surfactant protein D (SP-D)–and three non-organ-specific markers of systemic inflammation–high-sensitivity C-reactive protein (hsCRP), interleukin 6 (IL-6), and d-dimer.

Baseline serum samples prior to randomization were collected at SMART sites, centrifuged locally, then shipped to and stored at −70C at the study biospecimen repository (Advanced BioMedical Laboratories, Cinnaminson, NJ, USA). CC16 and SP-D levels were analyzed using commercially available sandwich ELISA kits (RD191022200 and RD194059101, BioVendor, Candler, NC, USA). hsCRP was measured using nephelometry (N High Sensitivity CRP; Siemens Healthcare Diagnostics, Deerfield, IL). IL-6 was measured by ultra-sensitive ELISA (Quantikine HS Human IL-6 Immunoassay; R&D Systems, Minneapolis, MN). D-dimer was measured by immunoturbidimetry (STA Liatest D-Di, STA-R analyzer, Diagnostica Stago, Asnières, France).

### Statistical Analyses

Baseline characteristics and biomarker levels were compared between cases and controls using conditional logistic regression models, accounting for the matching covariates of age, smoking, cART use and site of enrollment. An additional conditional logistic regression model included additional covariates of gender, race, HIV RNA, CD4+ count, prior AIDS, IV drug use and lipid-lowering drug use. Univariate logistic regression was used to compare biomarkers between smokers and non-smokers. In order to assess the ability of biomarkers to discriminate between case versus control status of a study participant, we also report the C-statistic (area under the receiver-operating characteristic [ROC] curve) for unconditional logistic regression models. All analyses were two-sided.

## Results

Among the 82 cases with bacterial pneumonia, 72 had stored blood available for this analysis. Most pneumonia cases had no organisms identified, but of the documented organisms, there were 6 cases of *Streptococcus pneumoniae*, 4 cases of *Legionella pneumophilia*, and 1 case each of Group B Streptococcus, *Staphylococcus aureus*, *Haemophilus influenzae*, and *Acinetobacter baumanii*. The median time from baseline study entry to pneumonia event was 17 months (IQR: 9–33 months). We were able to appropriately match all 72 cases to controls for a total sample size of 144 patients. Baseline demographic data for cases and controls are presented in Table 1. The two groups were similar with respect to baseline characteristics except for the bacterial pneumonia cases having a borderline higher proportion with prior AIDS (p = 0.06), lower proportion of lipid-lowering medication use (p = 0.06), and lower mean LDL level (p = 0.08).

**Table 1:** Baseline characteristics for SMART trial participants with a bacterial pneumonia event (cases) and controls matched on age, smoking status, baseline antiretroviral therapy use, and clinical center.

	Cases (n = 72)	Matched Controls (n = 72)	p-value^1^
Age, median years (IQR)	45 (41, 50)	45 (41, 52)	–
Gender (% female)	25.0	26.4	0.86
Race (% black)	51.4	51.4	0.99
Nadir CD4+ count, median cells/mm^3^ (IQR)	230 (124, 365)	257 (172, 353)	0.22
CD4+ count, median cells/mm^3^ (IQR)	519 (447, 635)	613 (436, 723)	0.30
HIV RNA ≤400 copies/mL (%)	52.8	55.6	0.68
Prior AIDS (%)	43.1	27.8	0.06
On antiretroviral therapy (%)	75.0	75.0	–
Antiretroviral therapy naive (%)	1.4	5.6	0.22
Co-infection with hepatitis B or C (%)	36.1	36.1	0.99
History of IDU (%)	18.1	16.7	0.81
Current smoker (%)	65.3	65.3	–
Diabetes (%)	8.3	11.1	0.59
Blood pressure lowering drugs (%)	29.2	23.6	0.42
Lipid lowering drugs (%)	6.9	18.1	0.06
Prior cardiovascular disease (%)	4.2	5.6	0.71
BMI, median kg/m^2^ (IQR)	25.6 (21.5, 29.7)	26.6 (22.9, 29.9)	0.24
Total cholesterol, median mg/dl (IQR)	169 (146, 207)	183 (158, 213)	0.10
HDL cholesterol, median mg/dl (IQR)	38 (33, 49)	42 (31, 54)	0.65
LDL cholesterol, median mg/dl (IQR)	94 (64, 123)	102 (81, 126)	0.08
Triglycerides, median mg/dl (IQR)	166 (97, 249)	159 (93, 299)	0.68
Total/HDL, median (IQR)	4.4 (3.2, 5.5)	4.6 (3.3, 6.2)	0.77

1 p-value from univariate conditional logistic model.

AIDS = acquired immunodeficiency syndrome, BMI = body mass index, HDL = high density lipoprotein, HIV = human immunodeficiency virus, IQR = interquartile range, IDU = injection drug use, LDL = low density lipoprotein, RNA = ribonucleic acid.

Comparison of biomarker levels between smokers (n = 94) and non-smokers (n = 50) (Table 2) showed that smokers had significantly lower CC16 levels than non-smokers, consistent with previous reports [17], [18]. SP-D, hsCRP, IL-6, and d-dimer levels were not statistically significantly different between smokers and non-smokers, although we had limited power for such comparisons. Among the 144 pooled cases and controls, there were no strong correlations between the five measured baseline biomarkers, with the exception of a modest correlation between hsCRP and IL-6 (r = 0.49, p<0.001) and d-dimer and IL-6 (r = 0.52, p<0.001)(Table 3).

**Table 2:** Median (IQR) levels of Surfactant Protein-D (SP-D), Club Cell Protein 16 (CC16), high-sensitivity C-reactive protein (hsCRP), Interleukin-6 (IL-6), and d-dimer levels by baseline smoking status (cases and controls combined).

	Smokers (n = 94)	Non-smokers (n = 50)	OR (95% CI)^1^	p-value^2^
CC16 (ng/mL)	4.1 (3.1, 6.4)	6.4 (4.6, 9.4)	0.5 (0.3–0.7)	<0.001
SP-D (ng/mL)	105 (69, 147)	87 (71, 120)	1.2 (0.8–1.9)	0.33
hsCRP (µg/mL)	2.37 (0.98, 5.43)	2.16 (1.12, 4.46)	1.2 (0.8, 1.8)	0.49
IL-6 (pg/mL)	2.36 (1.52, 4.40)	2.38 (1.32, 3.85)	1.1 (0.7, 1.7)	0.73
d-dimer (µg/mL)	0.30 (0.20, 0.59)	0.28 (0.17, 0.63)	0.9 (0.6, 1.4)	0.69

1 OR for smokers vs. non-smokers associated with a 1 IQR higher marker level after log_e_ transformation.

2 p-values from univariate logistic model.

**Table 3:** Spearman correlation coefficients (p-value) between baseline Club Cell Protein 16 (CC16), Surfactant Protein-D (SP-D), high-sensitivity C-reactive protein (hsCRP), Interleukin-6 (IL-6), and d-dimer for cases and controls combined.

	CC16	hsCRP	IL-6	d-dimer
**SP-D**	0.08 (0.37)	0.04 (0.66)	0.03 (0.71)	0.05 (0.59)
**CC16**		0.06 (0.50)	0.12 (0.15)	0.13 (0.13)
**hsCRP**			0.49 (<0.001)	0.16 (0.06)
**IL-6**				0.52 (<0.001)

For the primary analysis comparing baseline biomarker levels between cases and controls there was no statistical difference in SP-D or CC16 levels (Table 4). However, baseline hsCRP was significantly higher in cases than controls (2.94 µg/mL in cases vs. 1.93 µg/mL in controls; p = 0.02). IL-6 and d-dimer levels were also higher in cases compared to controls, although these comparisons did not meet statistical significance criteria (unadjusted p-values of 0.06 and 0.10, respectively). Adjusting for additional covariates did not alter these findings (Table 4).

**Table 4:** Median (IQR) biomarker levels in cases and controls.

	Cases (n = 72)	Controls (n = 72)	OR (95% CI)^1^	p-value^1^	AOR (95% CI)^2^	p-value^2^
CC16 (ng/mL)	5.0 (3.3, 6.8)	4.7 (3.5, 8.0)	1.1 (0.8–1.6)	0.54	1.2 (0.8–1.7)	0.40
SP-D (ng/mL)	93 (70, 144)	104 (68, 146)	1.0 (0.7–1.4)	0.98	0.8 (0.6–1.3)	0.42
hsCRP (µg/mL)	2.94 (1.37, 6.43)	1.93 (0.73, 3.58)	1.7 (1.1, 2.6)	0.02	1.9 (1.1, 3.2)	0.02
IL-6 (pg/mL)	3.34 (1.55, 5.98)	2.07 (1.31, 3.45)	1.5 (1.0, 2.4)	0.06	1.5 (1.0, 2.5)	0.08
d-dimer (µg/mL)	0.31 (0.22, 0.69)	0.25 (0.16, 0.50)	1.6 (0.9, 2.8)	0.10	1.5 (0.8, 2.7)	0.18

1 OR for bacterial pneumonia associated with a 1 IQR higher marker level after log_e_ transformation; matched variables include age, smoking, baseline cART use and site of enrollment.

2 OR for bacterial pneumonia associated with a 1 IQR higher marker level after log_e_ transformation adjusted for gender, race, HIV RNA, CD4+, prior AIDS, IDU and lipid lowering drugs (in addition to the matching variables age, smoking, baseline cART use and site of enrollment).

AOR = adjusted odds ratio, CC16 = Club Cell Protein 16, CI = confidence interval, hsCRP = high sensitivity C-reactive protein, IL-6 = interleukin 6, OR = odds ratio, SP-D = surfactant protein D.

ROC analyses of biomarker levels to discriminate the case versus control status of a given study participant showed that baseline covariates had a ROC area under curve (C-statistic) of 0.63 (Table 5). The addition of biomarker levels, alone or in combination, did not significantly improve the ability of the model to discriminate between the case versus control status of a given study participant.

**Table 5:** C-statistics for receiver-operating characteristic analyses of baseline covariates alone and with addition of baseline biomarkers.

Model	C-statistic	p-value
Baseline covariates, no biomarkers	0.63	-ref.-
Baseline covariates plus CC16	0.64	0.53
Baseline covariates plus SP-D	0.63	0.60
Baseline covariates plus hsCRP	0.69	0.11
Baseline covariates plus IL-6	0.68	0.18
Baseline covariates plus d-Dimer	0.67	0.17
Baseline covariates plus hsCRP and IL-6	0.69	0.12
Baseline covariates plus hsCRP and IL-6 and d-Dimer	0.69	0.12

p-values are derived from unconditional logistic regression models, and reflect the statistical significance of the difference of the C-statistic in biomarker-added models, compared to the baseline covariates (reference) model. CC16 = Club Cell Protein 16, hsCRP = high sensitivity C-reactive protein, IL-6 = interleukin 6, SP-D = surfactant protein D.

During our study, hsCRP, IL-6 and d-dimer levels were measured at baseline for all SMART participants. Therefore, while data were available only for a 1∶1 case:control ratio to study CC16 and SP-D, we were able to expand the number of controls for the hsCRP, IL-6, and d-dimer analyses. This was an unplanned post-hoc analysis. The resulting case:control mix of 72 cases and 238 controls resulted in similar odds ratios to our initial case:control ratio, but the added power resulted in lower p-values for hsCRP (OR 1.8; 95% CI:1.2–2.7; p = 0.006), IL-6 (OR 1.7; 95% CI:1.2–2.6; p = 0.005), and d-dimer (OR 1.5; 95% CI:1.0–2.2; p = 0.07).

## Discussion

We hypothesized that biomarkers of pulmonary inflammation would predict the development of bacterial pneumonia better than systemic markers of inflammation, but we found the opposite–hsCRP and IL-6 were related to bacterial pneumonia, but not CC16 or SP-D.

We studied CC16 and SP-D because they are proteins primarily produced in the lungs. CC16 is a 16 kDa protein produced by the non-ciliated club cells found in segmental bronchi, and CC16 is thought to play an anti-inflammatory role within the airways [19]. SP-D is a trimeric protein composed of three 43 kDa monomers and is secreted mainly by alveolar type II pneumocytes. In contrast to other surfactants that reduce alveolar surface tension, SP-D is believed to play an anti-inflammatory role at the alveolar level, while also playing a role in innate immunity [20]. Both CC16 and SP-D are secreted into the airways and alveoli where they exert their main functions, but levels are detectable in blood and become elevated in lung injury [21]–[23], supporting the potential use of these as non-invasive biomarkers of inflammation, injury, and epithelial integrity of the lung lining surfaces.

Our results suggest that baseline CC16 and SP-D have no relationship to bacterial pneumonia risk, at least in patients with HIV infection receiving effective cART. Among patients with chronic obstructive pulmonary disease (COPD), high serum SP-D levels are associated with an increased risk of acute exacerbations of COPD [24], most of which are triggered by bacterial or viral respiratory infections. However in this study, we were only able to assess for adjudicated bacterial pneumonia events and not more minor respiratory infections such as bronchitis episodes or viral and atypical pulmonary infections. We also note that these biomarkers may be susceptible to changes over time from a variety of pulmonary irritants [25]. Because we had only a single CC16 and SP-D measurement, we could not necessarily establish their stability, and this may have affected our results.

Our findings that baseline levels of the systemic inflammatory biomarkers hsCRP, IL-6, and d-dimer were associated with subsequent bacterial pneumonia risk are supported by previously published data. Among 3,075 community-dwelling elderly participants (mean age 73.6 years) followed for 6.5 years in the Health Aging and Body Composition cohort, higher baseline IL-6 levels were associated with a higher risk of developing subsequent community-acquired pneumonia [26]. Higher levels of plasma tumor necrosis factor, another marker of systemic inflammation, were also associated with an increased risk of pneumonia. CRP levels in that study were unrelated to pneumonia risk, though the CRP assay used in that study was not a newer high-sensitivity assay such as the one we used, so their CRP data may have been compromised to some degree. Two other studies of patients undergoing cardiac surgery also showed that elevated preoperative CRP levels were associated with increased postoperative respiratory infection risk [27], [28].

The mechanisms by which elevated systemic inflammatory markers are associated with increased bacterial pneumonia risk are not clear. Gram-negative bacteria express surface receptors for cytokines and in-vitro data have shown that IL-6, interferon gamma and other inflammatory cytokines promote bacterial growth in concentration-dependent fashion [29], [30], suggesting that these inflammatory markers may play a direct role in susceptibility to bacterial pneumonia. However, CRP has been shown to activate complement pathways and lead to opsonization of bacteria such as *Streptococcus pneumonia* [31], suggesting that CRP should perhaps be protective against pneumonia. Alternatively, systemic inflammatory markers may simply be markers of susceptibility rather than direct mediators of bacterial pneumonia risk. While we found no difference in co-morbid conditions associated with increased systemic inflammation such as diabetes or cardiovascular disease, we cannot exclude the possibility that elevations in systemic inflammatory markers might reflect other clinically unrecognized factors, such as lung disease, that render the host susceptible to pneumonia.

Underlying lung disease was not assessed as part of the SMART trial, so we could not compare nor adjust for conditions such as COPD present at baseline. Given that: 1) systemic inflammation is commonly observed in patients with COPD [32], 2) COPD is a risk factor for bacterial pneumonia [33], [34] and 3) HIV infection is an independent risk factor for COPD [35], we cannot exclude the possibility that the risk associated with elevated hsCRP, IL-6, and d-dimer levels we observed might reflect underlying lung disease. Recent data from HIV-infected patients demonstrates that ex-vivo stimulation of monocyte Toll-Like Receptor 2 (TLR2) upregulates inflammatory genes, including IL-6 pathways [36]. While gut microbial translocation is often cited as an etiology for TLR2 activation in HIV-infected patients, underlying lung disease such as COPD is also associated with TLR2 activation [37], possibly due to airway colonization with non-typeable *Haemophilus influenzae* [38].

We did note that prior AIDS was more common and lipid-lowering therapy use was lower in the bacterial pneumonia cases, compared to controls. Given that HMG-coA reductase inhibitors (i.e. statins) decrease hsCRP in the general population [39] and in patients with HIV infection [40], [41] and given that statins have been associated with possible decreased bacterial pneumonia risk [42], we included lipid-lowering therapy in a multivariate model. In this model, hsCRP continued to be higher in cases than controls, suggesting that the relationship between hsCRP and bacterial pneumonia is not simply due to hsCRP effects of statin use. Similar adjustment for prior AIDS did not change results.

Our study has several important limitations including a case:control design and limited sample size. While we matched on known confounders such as age and smoking status, and we adjusted for other potential confounders in our multivariate analyses, our analyses could still have been affected by other unrecognized confounders. While bacterial pneumonia was the most common clinical event reported in the SMART trial, it was still an infrequent event overall at 3.7% of participants over a mean follow-up of 32 months. While a larger case:control ratio for CC16 and SP-D would have increased the power for those analyses, the odds ratio point estimates of 1.2 and 0.8, respectively, suggest the lack of significant associations for these markers was not due to low power.

A major strength of our study is the adjudication of bacterial pneumonia events by a blinded endpoint review committee, rather than relying on clinician-diagnosed bacterial pneumonia or administrative data. As such, the cases were well defined. Our study population of treated participants with moderate/high CD4 counts and at low risk for AIDS is also a strength, in that they accurately represent contemporary patients in regions with access to cART. Other strengths of the study include the multi-center nature of the cohort and the close follow-up of patients as part of a clinical trial. Finally, we must acknowledge that while baseline levels of systemic inflammatory markers were higher in cases that eventually developed pneumonia compared to controls, our ROC analyses suggest that the use of these biomarkers in clinical decision-making appears limited.

In conclusion, among patients with HIV infection treated with continuous cART, patients with elevated baseline levels of systemic markers of inflammation appear to be at higher risk of developing bacterial pneumonia, and raises the question of whether treatments directed at reduction of systemic inflammation might reduce the risk of bacterial pneumonia. Two pulmonary-specific markers of lung inflammation, CC16 and SP-D, had no relationship to bacterial pneumonia. The mechanistic explanations for these observed relationships require further investigation.
